# Supplementary material for: A Simple Method for the Production of Human Skin Equivalent in 3D, Multi-Cell Culture
Source: Int J Mol Sci. 2020 Jun 30;21(13):4644. doi: 10.3390/ijms21134644 (PMC7369873; doi:10.3390/ijms21134644)
Supplement: Supplementary file 1 [file ijms-21-04644-s001.pdf]

Cells

Primary Epidermal Keratinocytes; Normal, Human, Neonatal Foreskin, HEKn (ATCC® PCS-200-010)

Primary Dermal Fibroblast Normal; Human, Neonatal, HDFn (ATCC® PCS-201-010)

Primary Epidermal Melanocytes; Normal, Human, Adult (HEMa) (ATCC® PCS-200-013)

Materials

Medium 106 (Gibco, cat# M106500);

Low Serum Growth Supplement, LSGS (Gibco, cat# S00310);

EpiLife Medium, with 60 µM calcium (Gibco, cat# MEPI500CA);

Human Keratinocyte Growth Supplement, HKGS (Gibco, cat# S0015);

10X Medium 199 (Gibco, cat# 11825015);

Collagen I Rat Protein, Tail (Gibco, cat# A1048301);

Calcium chloride, $CaCl_2$, CAS: 10043-52-4 (Merc, cat# 793639);

L-Ascorbic acid, CAS: 50-81-7, (Merc, cat# 50-81-7);

Genipin, (POL-AURA, cat# PA-03-8880-L-5G);

Keratinocyte Growth Factor, KGF (Gibco, cat# PHG0094);

10X Phosphate-buffered saline, 10X PBS (Gibco, cat# 70013073);

Penicillin-Streptomycin, P/S 10,000 U/mL (Gibco, cat# 15140122);

Sodium hydroxide, NaOH, CAS: 1310-73-2 (Merck, cat# 221465);

Fetal Bovine Serum, FBS (Gibco, cat# 10270106);

0.47 cm^2 Polycarbonate Cell Culture Inserts in Multi-Well Plates, 3-micron pores, (Nunc, cat# 140627);

24-well Carrier Plate (Nunc, cat# 141008).

Culture Medium Preparation

Fibroblast culture medium: Medium 106 supplemented with LSGS and P/S.

Keratinocyte culture: EpiLife Medium supplemented with HKGS and P/S.

Differentiation medium: EpiLife Medium supplemented with HKGS, P/S, 10% FBS, 110 µM $CaCl_2$, 10 ng/ml KGF, and 50 µg/ml L-Ascorbic acid.

Preparation of Human Skin Equivalents (Protocol 1).

To prepare 1 ml of fibroblast containing matrix:

1. Add 80 µl of 10X PBS, 113 µl of 10X Medium 199, 20 µl of 1 mM genipin, 17 µl of 1M NaOH and 670 µl of rat collagen I to a chilled tube.
2. Adjust pH to 6,8 – 7,2 if needed.
3. Add 2,5 x 10^5 of PCS-201-010 cells in 100 µl of fibroblast culture medium.
4. Mix by pipetting.
5. Place desired number of cell culture inserts in a carrier plate.
6. Add 400 µl of collagen solution to a cell culture insert and incubate in 37°C, 5% CO_2 for 60 min.
7. Fill the upper and lower chamber of the insert with fibroblast culture medium
8. Incubate the cell culture plate in 37°C, 5% CO_2 for 48-72h.
9. Carefully aspirate the medium from the outside and inside of the insert without touching the matrix.
10. Add 0,5 ml of keratinocyte culture medium to the outside of the insert.
11. Seed 2 x 10^5 of PCS-200-010 cells or PCS-200-010 mixed with 5-10% of PCS-200-013 cells onto the collagen scaffold in 200 µl of keratinocyte culture medium.
12. Incubate the cell culture plate in 37°C, 5% CO_2 for 48-72h.
13. Carefully aspirate the medium from the outside and inside of the insert without touching the matrix.
14. Using a sterile tweezer, place the culture inserts in the highest position in the carrier plate.
15. Add 1,5 ml of the differentiation media to the outside of the insert.
16. Incubate the cell culture plate in 37°C, 5% CO_2 for 2 to 3 weeks changing media every 2-4 days.

Preparation of histological slides (Protocol 2)

1. Remove the skin construct by carefully cutting the support membrane of the insert.
2. Using scalpel bisect the construct.
3. Place the bisected construct in a histology cassette and fix by 10% neutral buffered formalin for 24h.
4. Dehydrate and embed the construct in paraffin wax using a method of choice.
5. Using histological microtome, cut 5 µm sections of each embedded section and transfer onto microscope slides.
6. Incubate the slides in 60°C for 60 minutes.

Hematoxylin and Eosin Staining (Protocol 3)

Hematoxylin and Eosin Staining can be performed in automatic staining station or manually. For manual H&E staining:

1. Deparaffinize and rehydrate the sections by incubating the slides in:
    a. Xylene for 3 min (twice)

    b. 100 % ethanol for 3 min (twice)
    c. 95 % ethanol for 3 min
    d. 70 % ethanol for 3 min
    e. 50 % ethanol for 3 min
    f. Deionized water for 5 min
2. Stain with hematoxylin for 5 min
3. Rinse with tap water for 5 min
4. Dip 10 times in acid alcohol (70 % ethanol with 1 % HCl)
5. Rinse with tap water for 5 min
6. Stain with eosin for 30-60 sec
7. Rinse in tap water for 1 min
8. Dehydrate and clear the slides in:
    a. 95 % ethanol for 2 min (three times)
    b. 100 % ethanol for 2 min (three times)
    c. Xylene for 5 min (twice)
9. Mount using mounting media

Immunohistochemical Staining (Protocol 4)

1. Deparaffinize and rehydrate the sections by incubating the slides in:
    a. Xylene for 3 min (twice)
    b. 100 % ethanol for 3 min (twice)
    c. 95 % ethanol for 3 min
    d. 70 % ethanol for 3 min
    e. 50 % ethanol for 3 min
    f. Deionized water for 5 min
2. Retrieve antigens using 10mM sodium citrate buffer, pH 6.0
    a. Place slides in a Coplin jar filled with 10mM sodium citrate buffer, pH 6.0
    b. Place the Coplin jar in preheated to 95°C water bath and incubate for 25 min
    c. Let Coplin jar cool for 20 minutes at room temperature
3. Wash the slides for 5 min in TBS containing 0,025% Triton X-100
4. Block non-specific binding with TBS containing 5 % FBS and 1 % BSA for 2h
5. Drain slides
6. Add primary antibody or antibodies diluted (according to manufacturer recommendations) in TBS containing 1% BSA and incubate overnight at 4°C
7. Rinse with TBS containing 0,025% Triton X-100 for 5 min (twice)
8. If necessary, incubate in the dark, with secondary antibody diluted in TBS containing 1% BSA for 1 hour at room temperature
9. Rinse with TBS for 5 min (twice)
10. Mount using mounting media
